# Genome survey, high-resolution genetic linkage map construction, growth-related quantitative trait locus (QTL) identification and gene location in *Scylla paramamosain*

**DOI:** 10.1038/s41598-019-39070-z

**Published:** 2019-02-27

**Authors:** Ming Zhao, Wei Wang, Wei Chen, Chunyan Ma, Fengying Zhang, Keji Jiang, Junguo Liu, Le Diao, Heng Qian, Junxia Zhao, Tian Wang, Lingbo Ma

**Affiliations:** East China Sea Fisheries Research Institute, Chinese Academy of Fishery Sciences, 300 Jungong Road, Shanghai, 200090 China

## Abstract

*Scylla paramamosain* is one of the most economically important crabs in China. In this study, the first genome survey sequencing of this crab was performed, and the results revealed that the estimated genome size was 1.21 Gb with high heterozygosity (1.3%). Then, RAD technology was used to construct a high-resolution linkage map for this species. A total of 24,444 single nucleotide polymorphism (SNP) makers were grouped into 47 linkage groups. The total length of the linkage groups was 3087.53 cM with a markers interval of 0.92 cM. With the aid of transcriptome and genome scaffold data, 4,271 markers were linked to genes, including several important growth-related genes such as transforming growth factor-beta regulator I, immune related-gene C-type lectin and ecdysone pathway gene broad-complex-like protein. Further, 442 markers, representing 279 QTLs, associated with 24 traits were identified, and of these markers, 78 were linked to genes. Some interesting genes, such as dedicator of cytokinesis protein 3, tenascin-X and DNA helicase MCM8, were believed to have important relationship with specific traits and merit further exploration. The results of this study will accelerate the genetic improvement and genome sequencing analysis of the mud crab.

## Introduction

From phenotype breeding and molecular marker-assisted selection (MAS) to genome-wide selection (GS), artificial breeding strategies for economic animals have developed rapidly. Along with technological progress, artificial breeding has become increasingly effective, mainly through a more accurate enrichment of economically important quantitative traits and shorter breeding intervals. In 2001, Meuwissen *et al*.^1^ proposed the GS strategy and suggested that genome-wide dense markers should be used to predict genetic value. Further, the GS strategy has been demonstrated to be a popular tool for genetic improvement in livestock, and considerable achievements have been obtained, for example, dairy cattle^2^, chicken^3^ and pig^4^. However, in aquatic economic animals, especially crustacean species, mainstream breeding strategies in many species still consist of phenotype breeding and MAS. Other than unstable breeding and farming processes (compared with those of livestock), the lack of a high-quality genome and linkage map are two of the main problems that constrain the development of this industry.

With the revolution of sequencing technology, it is more effective to obtain a vast number of markers in a specific species, and thus, a high-resolution genetic linkage map becomes possible. Recently, several high-resolution genetic linkage maps were constructed in economic crustacean species, including *Penaeus monodon*^5^, *E. sinensis*^6^, *Litopenaeus vannamei*^7^, *Marsupenaeus japonicus*^8^, *Portunus trituberculatus*^9^. The average marker distances of these maps were all lower than 1 cM, which greatly assists *de novo* genome assembly and gene location, and further promotes the animal breeding process. Currently, the only crustacean genomes available are those of the water flea (*Daphnia pulex*)^10^, sand flea (*Parhyale hawaiensis*)^11^, marbled crayfish (*Procambarus virginalis*)^12^ and Chinese mitten crab (*Eriocheir sinensis*)^13^. Although most of these genomes were poorly assembled, they provided important insights into the genomic architecture and basic biology of crustacean species.

The mud crab *Scylla paramamosain*, belongs to the genus *Scylla* (Family: Portunidae), and is the most common mud crab in China^14^. As it is one of the most commercially important crustacean species, many research teams have been drawn to investigate the genetic breeding, aquaculture and basic biology of *S. paramamosain*. We have been devoted to studying the genetic breeding and aquaculture industry for many years, keeping ahead of the artificial breeding of this crab. Currently, there are no genome assembly datasets for this crab, and only several transcriptome libraries have been reported^15–19^. The only genetic linkage map for the mud crab was constructed by our team using 212 microsatellite and amplified fragment length polymorphism (AFLP) markers, and the mean spacing was 18.68 cM^20^. These data cannot meet the needs of the modern breeding strategies for this important species. Therefore, a high-resolution genetic linkage map is needed both the artificial breeding industry and genomics research.

The purpose of this study was to construct a high-resolution genetic linkage map for both QTL mapping, locating genes and assisting with further genome assembly and artificial breeding processes in *S. paramamosain*. Twenty-six traits of 99 F1 generation individuals were measured to obtain the trait-related loci. Genome survey data and transcriptome data were used in the gene location and growth gene identification analysis.

## Materials and Methods

### Ethics statement

All animal experiments in this study were conducted in accordance with the relevant national and international guidelines. Our project was approved by the East China Sea Fisheries Research Institute. In China, catching wild mud crabs from seawater does not require specific permits. Our study did not involve endangered or protected species.

### Genome survey sequencing and analysis

Genomic DNA of *S. paramamosain* was extracted from muscle for sequencing. Three pair-end DNA libraries, two with insert sizes of 400–500 bp and one with 250 bp, were constructed following standard Illumina operating procedures.

All raw data were trimmed to filter out low-quality data and adapter contaminant using NGS QC Toolkit^21^. *De novo* assembly was performed on the clean reads using SOAPdenovo software (http://soap.genomics.org.cn/soapdenovo.html) with the following parameters: the k value in k-mer was set at 45, unsolve repeats by reads and fill gaps in scaffolds.

### Mapping family and data collection

The F1 full-sib family for linkage map construction was created by two parents from the wild population of Hainan Province, China. The mapping population was reared at Wei-Er-Si Aquafarming Company of Rudong in 2017. In total, 99 progeny were randomly selected after being reared for 100 days in the same pond. Twenty-six traits, representing sex, carapace, cheliped, pereiopod and swimming stroke, were measured for each individual according to the measurement method described by Keenan *et al*.^22^. The measurement data are provided in the Supplementary Table 1.

Muscle tissues were sampled and preserved immediately in liquid nitrogen. Total DNA was extracted using a TIANamp Marine Animal DNA Extraction kit (TIANGEN, Beijing, China). DNA concentration was determined using GeneQuant (Amersham Biosciences Ltd., Piscataway, NJ, USA), and integrity was evaluated via electrophoresis in a 1% agarose gel.

### RAD library construction and Illumina HiSeq3000 sequencing

Restriction site-associated DNA marker (RAD) library construction, sample indexing and pooling followed for the natural populations^23^. The restriction enzyme EcoRI was used to cut the DNA. A total of 26 multiplexed sequencing libraries were constructed, in which each DNA sample was assigned a unique nucleotide multiplex identifier (MID) for barcoding. Pair-end (125-bp) sequencing was performed using Illumina HiSeq3000 in a total throughput of six lanes.

### SNP discovery, filtering, genotyping and validation

Raw sequence reads were trimmed to 110 nucleotides from the 3′ end to ensure that more than 98% of the nucleotides had a quality value above Q30 (equal to 0.1% sequencing error). The trimmed reads were clustered into read tags (hereafter, RAD-tags) by sequence similarity using ustacks^24^ to produce unique candidate alleles for each RAD locus. A maximum base-pair mismatch of one was allowed in this step for the genetic mapping population. RAD-tags were then collapsed into clusters using ustacks under the default parameters for SNP calling.

### Linkage map construction and QTL mapping

Genotype calling refers to the process of determining the genotypes of the SNP loci of each individual after SNP calling has been performed in accordance with Xu *et al*.^25^. Then, customized Perl scripts were applied to generate a “.loc” format file, which was the input file for joinmap 4.0^26^. The genetic map was grouped by joinmap 4.0 with logarithm of the odds (LOD) = 6.0 and the marker order was determined by Lep-MAP 2^27^. Singular markers were added to the established LGs using the joinSingles module with an LOD score limit of 10 and a minimum difference of 3 between the best LG and the second best LG of each joined marker. Then, markers intervals which are larger than 30 cM were removed, except for the LG32, in which the large interval might be caused by the location of centriole.

QTL mapping of the 26 traits was performed with MapQTL 5.0^28^. QTL region detection, the percentage of the phenotypic variance explained, and the genotypic information coefficient (GIC) were calculated with the interval QTL mapping model (IM)^29^. In the QTL mapping step, the LOD threshold for testing the significance of the QTL peaks was calculated using 1,000 permutations for each of the trait data sets and a genome-wide significant level of 5%. For interval distances >1.0 cM, significant thresholds were estimated every 1.0 cM. In this study, a LOD value of ≥3.0 was set as the minimum threshold to indicate a QTL in the present study.

### Gene location and growth-related gene identification

RAD-Tag sequences from the linkage map were BLASTed against genomic scaffolds and transcriptome unigenes, and a reference transcriptome was *de novo* assembled using the raw reads from our previous work^16,17^ and some unpublished data using the BLASTn program via an identity value cut-off of 99% and alignment length of 50. For alignment lengths between 30 and 50, manual inspection was performed. The transcriptome data, the genome scaffold data, and tag sequences from linkage map data referred to in this article are provided in Supplementary File 1. RAD-Tag sequences were also annotated via the GenBank nr database, which is hosted by NCBI (http://www.ncbi.nlm.nih.gov/), by blastx with an E-value cut-off of 1.0 × 10^−5^.

## Results

### Genome survey of *S. paramamosain*

Two 400- to 500-bp and one 250-bp paired-end libraries were constructed for the genome survey analysis, and 147.18 Gb of sequencing data were generated. Raw sequencing data were submitted to the Sequence Read Archive (SRA) database of NCBI with the accession number SRP150472. After QC and filtering, a total of 78.41 Gb of high-quality reads were obtained with a 45.32% GC content. The frequency of 17-mers (nucleotide strings with a length of 17 bp) among the raw sequencing data was calculated, and a k-mer curve was constructed. k-mer analysis revealed that there was a peak at the k-mer length of 60. The genome size was estimated at 1.21 Gb with remarkably high heterozygosity (1.3%), and 55.20% of the genome was estimated to be repetitive^30^. A total of 16,925,345 contigs with an N50 size of 145 bp were obtained, and of these sequences, 7,027,514 contained at least one simple sequence repeat (SSR), of which 3,900,204 were one-base repeats and 4,446,896 were two-base repeats.

### RAD sequencing and genotyping

There were 31 million high-quality reads for the female parent, and on average, 11 million for the offspring. The male parent could not be found because the female parent was captured from the open field sea. The female parent was sequenced at a higher depth (26.90×) than the mean depth of the offspring (8.37×). The statistics for the RAD-tag are summarized in Table 1.

**Table 1:** The statistical results of the RAD-tag for the female parents and the offspring.

Sample	Rad-tag number (depth >4)	Read length (bp)	Read number	Average depth (x)
Female	322,138	110	1,160,972	26.90
Mean of Mapping offspring	315,204	110	1,250,849	8.38

A total of 158,138 heterozygous polymorphic SNP markers were detected initially. A missed genotype number above nine on any SNP was filtered. After filtering, 29,069 high-quality SNP markers, that conformed to the expected Mendelian ratios (P ≥ 0.001) remained and were included in the linkage analysis.

### Linkage map construction

**Figure 1:**
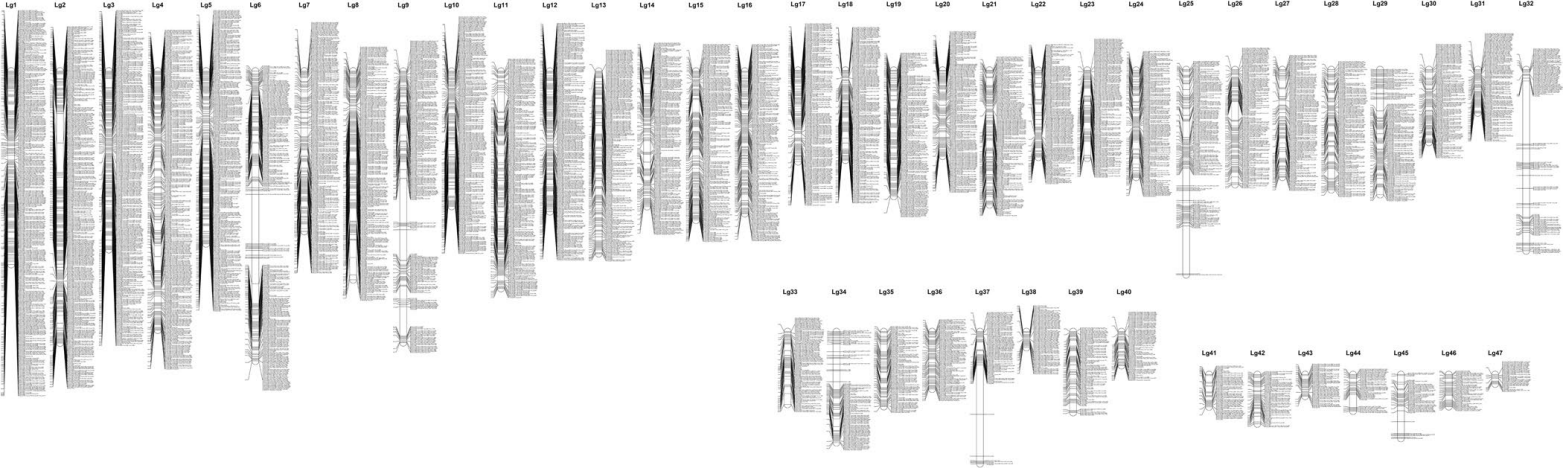
The 47 linkage groups of the high-density consensus linkage map of *S. paramamosain*.

At the LOD threshold of 6.0, 24,444 SNP loci were grouped into 47 linkage groups in the merged maps (Fig. 1), and 4,625 SNP markers could not be grouped. The 24,444 SNP markers contained three segregation types, including 13,261 lm x ll (54.3%), 9,125 nn x np (37.3%) and 2,058 hk x hk (8.4%). The genotype information for each SNP locus is provided in Supplementary Table 2, and the paternal information was derived from the maternal and F1 data. Maternal and paternal maps were also constructed, with 46 and 47 linkage groups, respectively. The lengths of the maternal, paternal and merged maps were 3,230.70 cM, 3,334.42 cM and 3,087.53 cM, respectively, and the average marker intervals were 1.87 cM, 1.82 cM and 0.92 cM, respectively. The length of the linkage groups in the maternal map varied from 6.12 cM (LG47) to 279.34 cM (LG2), and the length of those in the paternal map ranged from 6.19 cM (LG47) to 312.65 cM (LG8) (Table 2). The estimated genome size of *S. paramamosain* is 1.21 Gb; thus, the average recombination rate across all linkage groups was 2.55 cM/Mb.

**Table 2:** Summary of the consensus linkage map in *Scylla paramamosain*.

Linkage	Marker number	Paternal map	Maternal map	Consensus map	Estimated linkage group length of consensus map (cM)
1	1310	94.16	111.28	104.57	104.73
2	1194	141.49	279.34	146.97	147.22
3	1134	105.32	93.47	96.51	96.68
4	1096	100.69	104.52	140	140.26
5	998	123.58	124.17	93.35	93.54
6	857	176.09	144.47	156.76	157.13
7	841	81.25	161.85	86.68	86.89
8	819	312.65	113.61	112.78	113.06
9	814	204.58	118.48	146.72	147.08
10	802	83.61	81.4	73.07	73.25
11	777	91.41	140.31	117.41	117.71
12	774	70.71	63.63	75.95	76.15
13	661	97.12	71.64	98.64	98.94
14	638	101.08	57.01	73.19	73.42
15	611	111.54	114.48	80.51	80.77
16	608	73.88	79.53	76.98	77.23
17	603	61.72	45.2	49.42	49.58
18	584	53.68	51.85	48.57	48.74
19	540	69.52	61.45	68.15	68.40
20	539	38.04	42.03	46.42	46.59
21	487	72.91	72.62	72.75	73.05
22	478	53.61	42.29	47.02	47.22
23	477	58.79	35.98	47.56	47.76
24	468	66.39	56.98	57.85	58.10
25	455	74.16	77.44	110.85	111.34
26	448	66.08	63.06	62.11	62.39
27	445	39.63	36.04	58.13	58.39
28	422	68.22	74.38	64.9	65.21
29	384	59.04	69.67	67.55	67.90
30	376	41.23	38.03	39.3	39.51
31	365	17.35	27.75	23.4	23.53
32	330	50.12	59.05	97.94	98.54
33	322	37.42	39.33	39.32	39.56
34	284	60.09	62.36	60.03	60.45
35	267	30.76	67.2	40.49	40.79
36	266	31.85	27.72	31.07	31.30
37	259	103.35	94.5	71.41	71.96
38	254	17.35	0	8.63	8.70
39	254	36.29	53.37	43.71	44.06
40	229	18.54	19.47	18.34	18.50
41	181	17.77	19.85	17.52	17.71
42	162	37.49	22	26.62	26.95
43	146	14.39	10.2	11.72	11.88
44	129	12.31	30.69	19.77	20.08
45	129	35.64	46.1	34.45	34.99
46	121	15.33	20.78	17.35	17.64
47	106	6.19	6.12	5.09	5.19
Sum	24444	3334.42	3232.70	3087.53	3087.78

### QTL mapping

**Figure 2:**
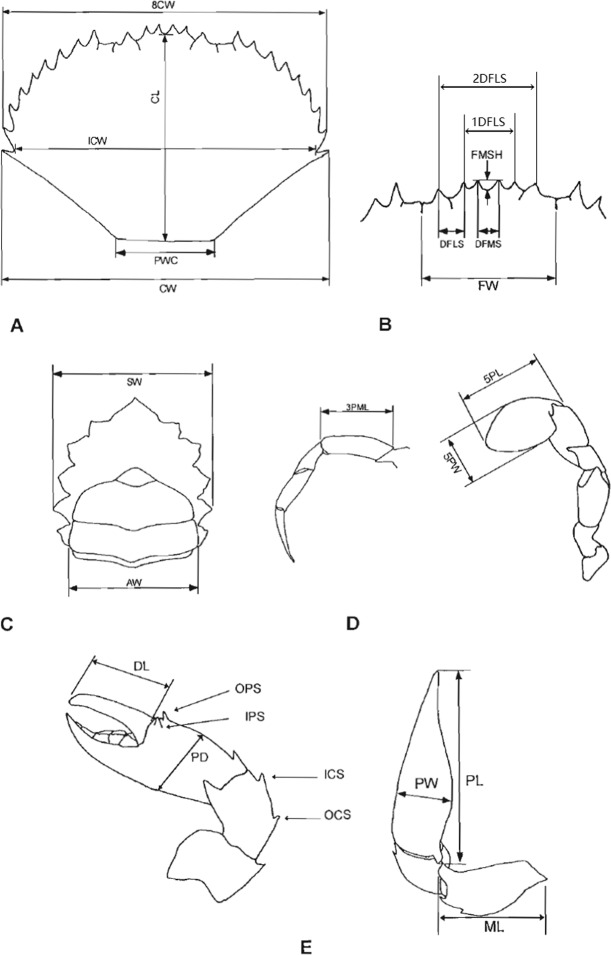
Measurements traits of *S. paramamosain*^21^. (**A**) Carapace, (**B**) frontal lobe, (**C**) sternum, (**D**) periopods and (**E**) chelipeds. Appearance size: carapace length (CL), carapace width (CW), carapace width at spine 8 (8CW) and body height(BH); head area: carapace frontal width (FW), distance between frontal median spines (DFMS), frontal median spine height (FMSH), distance between frontal lateral spines(DFLS), distance between the first frontal lateral spines (1DFLS), distance between the second frontal lateral spines (2DFLS); cheliped: dactyl length (DL), merus length (ML), propodus length (PL), propodus width (PW), propodus depth (PD); Periopod and swimming stroke: 1st periopod merus length (1PML), 2nd periopod merus length (2PML), 3st periopod merus length (3PML), swimming stroke dactyl width (5PW), swimming stroke dactyl length (5PL); Reproductive ability: abdomen width (AW).

The 26 traits of the 99 mapped filial individuals, which included 78 females and 21 males, are listed in Supplementary Table 3. In total, 442 SNP loci, with a LOD > 3, which representing 279 QTLs, corresponded to 24 traits. We divided these 24 traits into five categories: appearance size, head area, cheliped, pereiopod and swimming stroke, and reproductive ability (Fig. 2). The LOD values of these 442 loci ranged from 3.01 to 12.66. Five markers with a LOD value greater than five were associated with seven traits, including three cheliped traits (markers 11.hk_hk_10468 and 11.hk_hk_11304), three pereiopod and swimming stroke traits (markers 11.hk_hk_7558, 105.nn_np_34985 and 11.hk_hk_11304) and one size trait (11.nn_np_24679). Among the 442 markers, 36 markers were linked to more than one trait, suggesting that these traits might be under the same genetic control. Two to 58 markers distributed in one, two, three or four linkage groups were linked to 24 traits. SW had the most linked markers (58), followed by 2PML (55), 3PML (48), and FW (44), whereas CW and ML had only two markers. The markers associated with the 24 traits are summarized in Table 3.

**Table 3:** Summary of the 24 traits associated QTLs.

Traits	Sex	OCS	CL	CW	8CW	AW	SW	BH
QTL number	12	15	5	2	8	4	58	4
LG-QTL number	10–7; 9–3; 12–2	30–13; 14–1; 29–1	12–3; 41–2	29–2	11–8	11–3; 10–1	9–26; 40–24; 34–8	2–4
**Traits**	**FW**	**DFMS**	**FMSH**	**DFLS**	**1DFLS**	**2DFLS**	**DL**	**ML**
QTL number	44	37	7	33	32	17	3	2
LG-QTL number	25–43; 28–1	15–18; 12–16; 25–2; 34–1	17–7	13–33	6–26; 28–4; 2–2	11–16; 2–1	15–1; 22–1; 26–1	14–1; 26–1
**Traits**	**PL**	**PW**	**PD**	**1PML**	**2PML**	**3PML**	**5PL**	**5PW**
QTL number	4	6	24	31	55	48	17	25
LG-QTL number	14–2; 11–2	13–4; 14–2	5–15; 26–6; 3–3	25–28; 11–1; 14–1; 16–1	2–30; 28–15; 13–9; 35–1	12–26; 5–21; 14–1	6–9; 25–7; 14–1	25–24; 14–1

**Figure 3:**
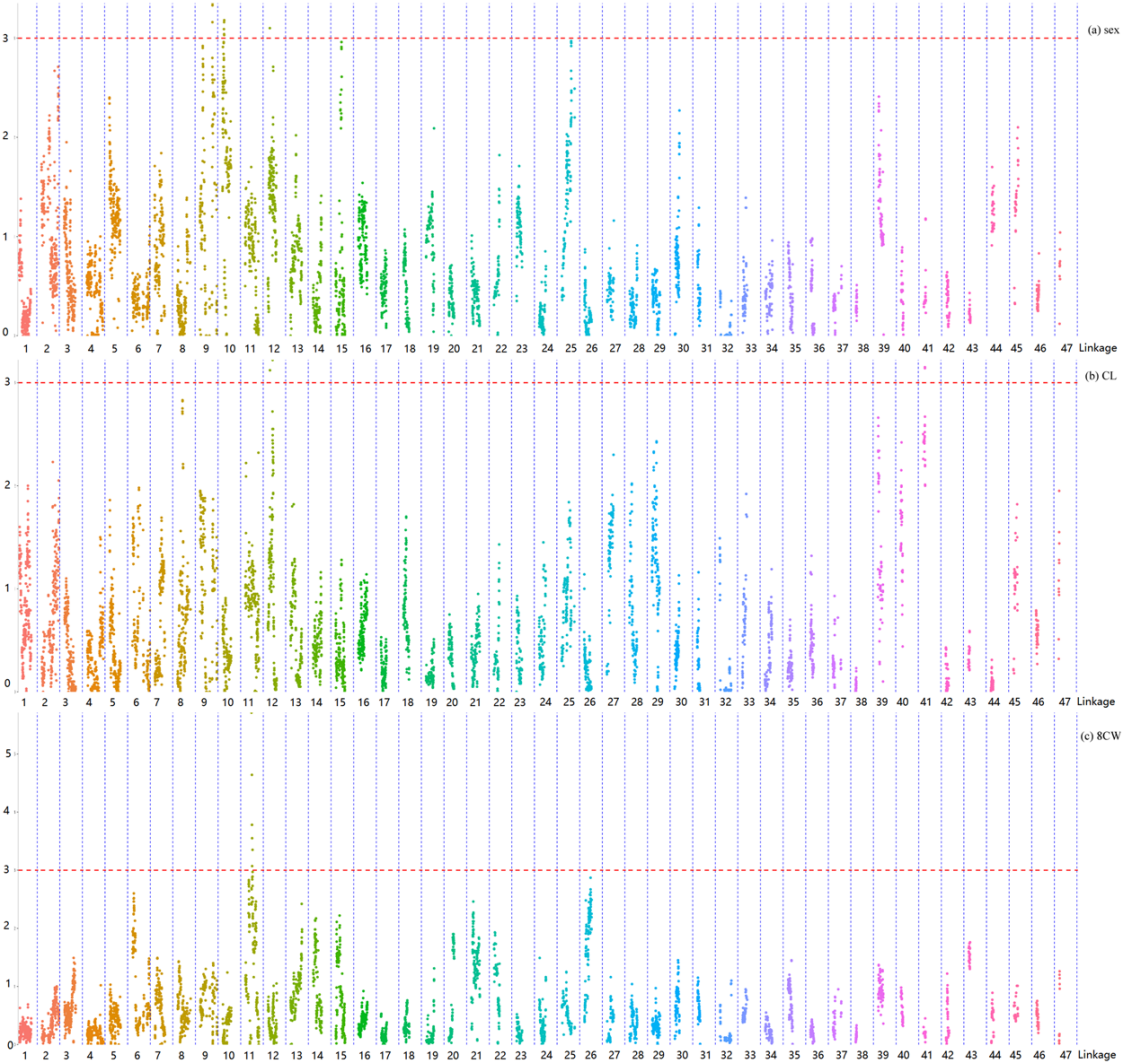
QTL mapping for traits including sex (**a**), carapace length (CL) (**b**) and carapace width at spine 8 (8CW) (**c**).

Twelve markers were linked to sex and distributed in LG10 (7), LG12 (2) and LG9 (3) (Fig. 3a). CL (Fig. 3b), CW, 8CW (Fig. 3c) and BH, four important traits representing the appearance size of the crab, were linked to 19 markers distributed in LG11 (8), LG2 (4), LG12 (3), LG29 (2) and LG41 (2). Five traits (DL, ML, PL, PW and PD), indicating the developmental properties of the cheliped, were linked to 37 markers distributed in LG5 (15), LG26 (8), LG13 (4), LG14 (3), LG3 (3), LG11 (2), LG15 (1) and LG22 (1). Five traits (1PML, 2PML, 3PML, 5PW and 5PL) associated with pereiopod and swimming stroke had 168 markers distributed in 11 linkage groups, represented by LG25 (52), LG2 (30), LG12 (26) and LG5 (21). Six traits that might correlate with the development of the head area (FW, DFMS, FMSH, DFLS, 1DFLS and 2DFLS), had 168 markers distributed in 10 linkage groups, represented by LG25 (43), LG13 (33), LG6 (26) and LG15 (18). AW might have some correlation with reproductive ability, with four markers located in LG11 (3) and LG10 (1).

### Gene location and growth gene identification

By means of BLASTing against the nr database, transcriptome and scaffold of the genome survey, 4,271 of the linkage map markers were linked to genes, and the gene numbers distributed in each linkage group ranged from 15 to 210. LG2 has the largest number of located genes, followed by LG3 (204), LG1 (198), LG11(180) and LG4 (171). LG47 has the smallest number of genes, followed by LG44 (16), LG45 (20), LG46 (32), LG41 (37) and LG43 (38) (Supplementary Table 4). Gene numbers were generally positively correlated with the length and markers of linkage groups, with correlation coefficient of 0.818 and 0.823, respectively. Some growth-related genes, including transforming growth factor-beta regulator I, insulin-like androgenic gland hormone and insulin-like growth factor 2 mRNA-binding protein, were located. C-type lectin, which participates in the immune defense process, HR4 nuclear receptor and broad-complex-like protein, which participates in the ecdysone regulation pathway, were also located.

In total, 78 markers have been linked to genes. Among them, 22 markers had annotation information from at least one public database, and others were linked to transcriptome data but lacked annotation information. Of these 22 markers, two were located in the 3′-UTR, seven were in the intron, 11 were in the open reading frame (ORF), and four led to a nonsynonymous mutation; the other two were located in a pseudogene because an abnormal termination codon existed in the translated region (Table 4). 11.hk_hk_4825, a synonymous SNP located in the ORF of a RING finger protein, was a QTL of FW and 5PW. Two RING finger proteins, nhl-1, nesprin-2-like, RNA-directed DNA polymerase from mobile element jockey-like, dedicator of cytokinesis protein 3 isoform X2, carbonic anhydrase 2-like and protein disulfide-isomerase A5-like each had one SNP marker associated with FW (four), DFMS (two) and 2DFLS (one), and these genes or their linked genes might be related to the development of head area. Furthermore, integrator complex subunit 8-like, tenascin-X-like, sialidase-like, BTB/POZ domain-containing protein 3, polypeptide N-acetylgalactosaminyl transferase 1 and cholinephosphotransferase 1 isoform or their linked genes might be related to pereiopod and swimming stroke development. Mitochondrial ribonuclease P protein 3, sedoheptulokinase-like and putative nuclease HARBI1 are three genes with SNP associated with cheliped related traits, and them or their linked genes might be related to cheliped development. In addition, ankyrin repeat and general transcription factor II-I repeat domain-containing protein 2B-like or their linked genes might be related to the appearance size of the crab. GPI mannosyltransferase 4, DNA helicase MCM8-like and kinesin II or their linked genes might be related to reproductive ability, and protein numb isoform X5 or its linked genes might be related to OCS.

**Table 4:** Statistics of growth-related genes.

Category	Marker ID	Linkage Group	Position (cM)	Traits	Annotation	Marker location
Head area	118.nn_np_13921	Lg15	39.65	DFMS	dedicator of cytokinesis protein 3 isoform X2	Intron
	118.lm_ll_17019	Lg15	38.63	DFMS	carbonic anhydrase 2-like	ORF (nonsynonymous) Ser to Asn
	102.nn_np_15059	Lg25	43.24	FW	RING finger protein nhl-1	Intron
	10.lm_ll_3905	Lg25	40.19	FW	nesprin-2-like	ORF (synonymous)
	11.hk_hk_4825	Lg25	64.36	FW	RING finger protein NHL-1/tripartite motif-containing protein 2-like/E3 ubiquitin-protein ligase TRIM71	ORF (synonymous)
	10.nn_np_29892	Lg25	44.76	FW	RNA-directed DNA polymerase from mobile element jockey-like	ORF (synonymous)
	105.lm_ll_5318	Lg11	109.71	2DFLS	protein disulfide-isomerase A5-like	ORF (synonymous)
Pereiopod and swimming stroke	10.lm_ll_12123	Lg25	20.57	1PML	cholinephosphotransferase 1 isoform	ORF (synonymous)
	105.lm_ll_21946	Lg5	15.76	5PL	sialidase-like	ORF (nonsynonymous) Ser to Asn
Cheliped	11.nn_np_19207	Lg26	52.84	ML	mitochondrial ribonuclease P protein 3	ORF (nonsynonymous) Trp to Arg
	10.nn_np_21822	Lg26	11.19	PD	putative nuclease HARBI1	pseudogene
	105.nn_np_19997	Lg22	44.99	DL	sedoheptulokinase-like	ORF(synonymous)
Appearance size	11.nn_np_8382	Lg41	15.49	CL	general transcription factor II-I repeat domain-containing protein 2B-like	pseudogene
	118.nn_np_30643	Lg11	58.04	8CW	Ankyrin repeat	Intron
Reproduction ability	118.lm_ll_9231	Lg40	9.16	AW	kinesin II	ORF (synonymous)
	103.nn_np_11565	Lg34	0	AW	GPI mannosyltransferase 4	Intron
	11.lm_ll_10814	Lg40	3.56	AW	DNA helicase MCM8-like	Intron
OCS	11.hk_hk_3949	Lg30	33.69	OCS	protein numb isoform X5	Intron

## Discussion

Mud crabs are euryhaline and widely distributed in tropical, subtropical and temperate waters. In China, *S. paramamosain* is the dominant species and is mainly cultured in southeastern coastal provinces^14^. Mud crabs are very popular in China because of their high content of protein, unsaturated fatty acids and trace elements such as vitamins^31^.

Genome sequencing is an important step for deciphering evolutionary status and molecular mechanisms and accelerating genetic improvements in traits of interest in economically important species. The genome size of *S. paramamosain* was estimated to be 1.21 Gb with remarkably high heterozygosity (1.3%). This finding is inconsistent with previous studies (1.64 pg), in which flow cytometry was used to assess genome size^32^. Different results for one species from two methods were also found in *P. trituberculatus*^9^ and *L. vannamei*^7^. The flow cytometry method is fairly straightforward, but the accuracy is highly dependent on the internal standard and quality of the material used for DNA content measurement^33^. Additionally, a comparison between flow cytometry and k-mer analysis methods in the estimation of nine insect species suggested that k-mer analysis is more accurate^34^. Genome survey analysis will be highly useful for the formulation of sequencing strategies for the mud crab.

Currently, the only reported linkage map of this species was constructed by our team using microsatellite and AFLP markers. In Total, 212 markers were mapped, and the mean spacing of the markers was 18.68 cM^20^, which cannot fulfill the requirements of the genetic breeding industry. This study reported a high-resolution genetic map of the mud crab with 24,444 SNP markers, a length of 3087.53 cM and a marker interval of 0.92 cM. This map has the highest number of markers compared with that of maps of other crustacean species, including *P. trituberculatus* (10,963 markers)^9^, *M. japonicus* (9289 markers)^8^ and *L. vannamei* (6146 markers)^7^. A large number of markers in the map were at the same location, which means they are completly linked. Therefore, the marker interval should be 0.92 cM instead of 0.13 cM (3087.53/24,444≈0.13). However, the numerous markers will be very useful in QTL mapping, gene location and genome assemble.

Forty-seven linkage groups were obtained in the merged map, and only 46 linkage groups were in the maternal map. The missing linkage group in the maternal map was LG38; this linkage group may not be the sex chromosome because alleles in this linkage were not separated significantly by sex in the F1 offspring. Forty genes were located on LG38, and no sex-determining genes were found in this linkage group. A high-density linkage map suggested a ZW sex determination system in *Eriocheir sinensis*; but only an *ankyrin-2* gene was found on the putative sex chromosome, and the sex determination gene *double-sex* was located on a putative autosome^6^. This finding suggested that sex determination genes may not exist on sex-linked chromosomes or that there is a different sex determination system in crustaceans. Further, 12-sex linked QTL were identified, and verification in large numbers of wild populations is needed.

Under the conditions of high marker density (24,444 SNPs), the linkage groups should be consistent with the haploid chromosome number. However, the number of linkage groups in the present map seems to be less than the 49 haploid chromosome numbers reported by Wang *et al*.^35^ and Chen *et al*.^36^ using karyotype method. In fact, fewer number of linkage groups in the linkage map were also found in *M. japonicus*^8^. In this study, the lack of one linkage group in the maternal map and the lower numbers in the merged map might be mostly due to the relatively lower number of mapping populations or the low enzyme-digestion efficiency in the missing chromosome.

Overall, 4,271 markers were linked to genes, and the gene numbers were generally positively correlated with the length and marker numbers of linkage groups, suggesting that genes were almost evenly distributed in the crab genome. Transforming growth factor-beta regulator I, which is also called nuclear interactor of ARF and Mdm2 (NIAM), activates p53/TP53, causes G1 arrest and collaborates with ARF to suppress proliferation, acted as a growth inhibitor^37^. Insulin-like androgenic gland hormone (IAG), which is produced by androgenic glands in male crustaceans, is regarded as a key regulator of sex differentiation. Interestingly, studies on IAG in *Scylla paramamosain* suggested that it is involved in regulating ovarian development and somatic growth^38^. Insulin-like growth factor 2 mRNA-binding protein binds to the 5′-UTR of the insulin-like growth factor 2 (IGF2) mRNA to regulate IGF2 translation, and thus has an important function in animal development^39^. C-type lectins are a superfamily of proteins that recognize a broad repertoire of ligands and regulate a diverse range of physiological functions^40^. Indeed, C-type lectins of *S. paramamosain* were upregulated significantly by *Vibrio parahaemolyticus* and had an important role in the immune response^41,42^. Broad-complex-like protein and HR4 nuclear receptor are two ecdysteroidogenic transcription factors that deliver ecdysone signal, which is also important for development^43^. Knowing the location of these genes will assist the study of genetic breeding.

The head area of the crab contains many organs, such as the cerebral ganglion and antennary glands. Seven linked annotated genes were associated with this area. Among them, nesprin-2 belongs to a novel family of nuclear and cytoskeletal proteins with rapidly expanding roles as intracellular scaffolds and linkers^44^. Dedicator of cytokinesis protein 3 (DOCK3) is a large protein involved in intracellular signalling networks, which function as activators of small G proteins. DOCK3 is expressed exclusively in the central nervous system of mice and plays an important role in axonal outgrowth and cytoskeleton re-organization^45^.

Six genes associated with pereiopod and swimming stroke traits were identified. Among them, tenascin-X is a glycoprotein that contributes to matrix stability and is possibly involved in collagen fibril formation^46^. A SNP in this gene causes a nonsynonymous mutation, which replaces a hydrophilic amino acid Ser with a hydrophobic amino acid Leu. Sialidase-like, which is a glycoside hydrolase enzyme that cleaves the glycosidic linkages of neuraminic acids^47^, also possesses a nonsynonymous mutation of Ser to Asn. Future studies in large populations are needed to prove the correlation between phenotype and genotype. Polypeptide N-acetylgalactosaminyltransferase 1, which catalyses the initial reaction in O-linked oligosaccharide biosynthesis^48^, and cholinephosphotransferase 1, which catalyses phosphatidylcholine biosynthesis from CDP-choline and plays a central role in the formation and maintenance of vesicular membranes^49^, were also suggested to have a relationship with pereiopod and swimming stroke development.

Three linked annotated genes were suggested to associate with cheliped traits. Among them, mitochondrial ribonuclease P protein 3, which is a part of mitochondrial ribonuclease P (mt-RNase P) that cleaves tRNA molecules at their 5′ -ends^50^, and a nonsynonymous mutation of Trp to Arg was found in this gene. In addition, the putative nuclease HARBI1, which might promote anti-*V. alginolyticus* infection by participating in regulating phagocytosis, apoptosis, superoxide dismutase activity, PO activity, and THC in *Marsupenaeus japonicus*, was another gene associated with cheliped traits^51^.

The ankyrin repeat was linked to one QTL of 8CW and appeared to be associated with appearance size. Proteins that contain the ankyrin repeat motif can mediate the protein-protein interactions^52^.

Beneath the abdominal plate is the location in which the eggs are held in female crabs and where the ejaculatory duct is found in male crabs, both of which were possibly associated with reproductive ability. Three genes were linked to QTLs of AW, among which GPI mannosyltransferase 4 is involved in glycosylphosphatidylinositol-anchor biosynthesis^53^. DNA helicase MCM8, forms a complex with MCM9 and is involved in homologous recombination repair following DNA interstrand cross-links, thus playing a key role during gametogenesis^54^.

It is likely that some of the association genes seem to have some relationship with specific traits, which indicated by their function studies in other species. However, it should be noted that the association genes do not mean that they are the causative genes, and in-depth studies are still needed.

In summary, this study performed a genome survey analysis, which revealed that *S. paramamosain* has an estimated genome size of 1.21 Gb with high heterozygosity (1.3%). Then, a high-resolution linkage map was constructed using 24,444 SNP makers. Forty-seven linkage groups were obtained with a total length of 3087.53 cM and a marker interval of 0.92 cM. With the aid of transcriptome and genome scaffold data, 4,271 markers were linked to genes, including some important growth-related, immune-related and hormone pathway genes. Further, 442 QTLs were identified and found to correspond to 24 traits, and of these, 78 QTLs were linked to genes. Some interesting genes, such as dedicator of cytokinesis protein 3, tenascin-X and DNA helicase MCM8, were believed to have important relationships with specific traits and are worth further exploration.

## Supplementary information

Dataset 1

Dataset 2

Dataset 3

Dataset 4

Dataset 5
